# *Festuca pratensis*-like Subgenome Reassembly from a “Chromosomal Cocktail” in the Intergeneric *Festulolium* (Poaceae) Hybrid: A Rare Chromoanagenesis Event in Grasses

**DOI:** 10.3390/plants12050984

**Published:** 2023-02-21

**Authors:** Izolda Pašakinskienė

**Affiliations:** 1 Life Sciences Centre, Vilnius University, Saulėtekio 7, 10221 Vilnius, Lithuania; izolda.pasakinskiene@gf.vu.lt; 2 Botanical Garden of Vilnius University, Kairėnų 43, 10239 Vilnius, Lithuania

**Keywords:** hybrid instability, chromosome rearrangements, genome plasticity, 45S rDNA, FISH, GISH

## Abstract

*Festuca* and *Lolium* grass species are used for *Festulolium* hybrid variety production where they display trait complementarities. However, at the genome level, they show antagonisms and a broad scale of rearrangements. A rare case of an unstable hybrid, a donor plant manifesting pronounced variability of its clonal parts, was discovered in the F2 group of 682 plants of *Lolium multiflorum* × *Festuca arundinacea* (2n = 6x = 42). Five phenotypically distinct clonal plants were determined to be diploids, having only 14 chromosomes out of the 42 in the donor. GISH defined the diploids as having the basic genome from *F. pratensis* (2n = 2x = 14), one of the progenitors of *F. arundinacea* (2n = 6x = 42), with minor components from *L. multiflorum* and another subgenome, *F. glaucescens*. The 45S rDNA position on two chromosomes also corresponded to the variant of *F. pratensis* in the *F. arundinacea* parent. In the highly unbalanced donor genome, *F. pratensis* was the least represented, but the most involved in numerous recombinant chromosomes. Specifically, FISH highlighted 45S rDNA-containing clusters involved in the formation of unusual chromosomal associations in the donor plant, suggesting their active role in karyotype realignment. The results of this study show that *F. pratensis* chromosomes have a particular fundamental drive for restructuring, which prompts the disassembly/reassembly processes. The finding of *F. pratensis* “escaping” and rebuilding itself from the chaotic “chromosomal cocktail” of the donor plant points to a rare chromoanagenesis event and extends the view of plant genome plasticity.

## 1. Introduction

In the plant kingdom, cross-hybridization between species is a rather common event, which in many instances results in the establishment of new allopolyploid taxa [1,2,3]. Polyploids are also frequent in domesticated and economically important crops [4,5,6]. It has been widely demonstrated that the newly formed allopolyploids are more than just the sum of their parents and that they have gone through complex processes of genome adjustment. Hybridization challenges the integrity of genomes and leads to a cascade of structural changes, and these responses are species- and genotype-dependent [7,8,9,10,11,12,13]. Studies in the lineages of nascent allopolyploids show numerous rearrangements described as biased fractionation [4], subgenome dominance [14,15], karyotype reshuffling [16,17], and genomic mosaicism [18].

In plants, new individuals can arise not only in a sexual way (through meiosis, gametes, fertilization), but somatic clones (through mitosis) are also readily available in vitro and in vivo. Grasses have a capacity for tillering; new tillers emerge at basal nodes, a small zone between the root and the stem. During their life, plants, and distant hybrids especially, accumulate somatic irregularities at the level of cell populations in their body, which further leads to the development of chimeric clones genetically differing from the donor plant [19,20]. *Saccharum* spp. and *Phalaris* spp. are classical examples of clonally propagated species where ploidy changes and chromosome structure alterations have been widely recorded [21,22].

In Poaceae, *Lolium* and *Festuca* genera are considered to have diverged from the common ancestor that had a basic chromosome number of x = 7 [23,24]. Several *Lolium* and *Festuca* species are very closely related. They hybridize easily, and the crosses between *L. multiflorum* and diploid *F. pratensis*, or hexaploid *F. arundinacea*, demonstrate particularly high compatibility, providing the germplasm for the development of commercial grass varieties [25,26,27,28]. *F. arundinacea* (2n = 6x = 42) is a natural allohexaploid species originating as a hybrid between *F. pratensis* and *F. glaucescens*, structurally defined as FpFpFgFgFgFg [29]. In addition, *L. multiflorum* was found to be the third species involved in the development of *F. arundinacea* by its introgression within the *F. pratensis* subgenome [30]. Moreover, this study has shown that there is much closer homology between *L. multiflorum* and *F. pratensis* genomes than between *L. multiflorum* and *F. glaucescens*. In grass breeding, *L. multiflorum* and *F. arundinacea* show very high crossability and provide a particularly valuable combination of complementary traits [28,31,32].

In brief, there are two groups of *Lolium* × *Festuca* hybrids, tetraploids (2n = 4x = 28) and hexaploids/octoploids (2n = 6x = 42/2n = 8x = 56). *L. multiflorum* × *F. arundinacea* hexaploids/octoploids have been less studied in comparison to other *Festulolium* hybrids, and tendencies of DNA content reduction (downsizing) and ploidy level change were found both in early F1 and later generations of these hybrids [33,34,35]. In these high ploidy *L. multiflorum* × *F. arundinacea* (2n = 8x = 56) alloctoploids, the phenomenon of somatic instability manifesting itself by instant rediploidization was previously discovered in colchicine doubled F1C0 [36].

In this study, we follow up on the story of rediploidization in the next F2C1 progeny, concentrating on a particular “super-recombinant” *L. multiflorum* × *F. arundinacea* (2n = 6x = 42) hexaploid genotype characterized as a “chromosomal cocktail”. We reveal the changes and trace them from plant morphology going deeper into the karyotype/genotype level. Once again, as in F1, we record diploid plants in F2 as a result of the split allohexaploid genome in clonal plants and show these “escapees” to be close to the *F. pratensis* subgenome of *F. arundinacea*.

Summarizing the results, we discuss a possible scenario of the re-establishment of diploid segregants occurring throughout the hybrid genome response that could reach the status of unpredictable chromosome shattering and reassembly known under the term chromoanagenesis. Chromoanagenesis was first defined by Holland and Cleveland [37] from the “cellular cataclysm” studies observed in human cancer research. Chromoanagenesis (from *chromo* for chromosomes and *anagenesis* for “rebirth”) is a phenomenon in which large numbers of complex rearrangements occur involving one or a few chromosomes in a single catastrophic event [37,38,39]. So far, there are only a few instances of chromoanagenesis described in plants, in grapevine [40] and *Populus* [41]. The results of this study add to the conceptual ideas on how dramatically genomes can rearrange themselves when fused together in intergeneric hybrids.

## 2. Materials and Methods

### 2.1. Plant Material

**Figure 1:**
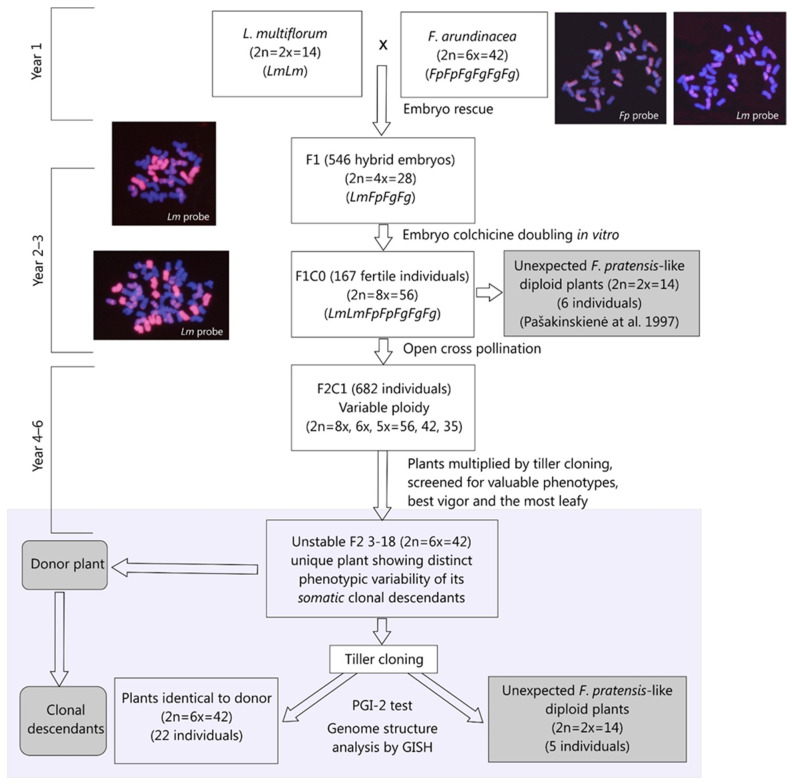
The scheme of the crosses and clone pedigree, together with the GISH images for the parental *F. arundinacea* and F1C0 and F2C1 hybrids, demonstrate the specificity of the *L. multiflorum* rhodamine labelled probe for highlighting simultaneously *L. multiflorum* (red) and *F. pratensis* (mauve) chromosomes. Abbreviations: Lm—*L. multiflorum*, Fp—*F. pratensis*, Fg—*F. glaucescens* (Fp and Fg are subgenomes comprising *F. arundinacea* parent). The experimental plot of this study is highlighted in light gray.

The pedigree and experimental chronology are shown in Figure 1. To produce F1 hybrids, the anthers were removed from diploid *L. multiflorum* (2n = 2x = 14) plants, var. ‘Prima Roskilde’ and var. ‘S.22’. The inflorescences were bagged and pollinated with the pollen of hexaploid *F. arundinacea* var. ‘Barundi’ genotype no. 870. The hybrids required embryo rescue; they were isolated after 16–18 days and grown on LS medium. The germinated embryos were treated with 0.3% colchicine solution, and F1C0 hybrids were obtained [36]. One hundred sixty-seven F1C0 fertile plants were planted in an isolation plot to allow for cross-pollination and seed setting. From the bulk of seeds obtained, a group of 682 F2C1 plants was grown in the experimental field of The Lithuanian Institute of Agriculture. Plants were grown at a distance of 50 × 50 cm and evaluated visually for their growth habits, tiller number, and width and length of their leaves. This way, a particular F2 3-18 hybrid, the donor plant, showing distinct tiller chimerism was detected. The F2 3-18 plant was split by a single tiller, and 27 individual plants were established. Five distinct clonal descendants were specified, each having a different growth habit and leaf shape than the donor plant. The donor plant and clonally obtained individuals were used in this study.

### 2.2. Phosphoglucoisomerase-2 Isozyme Assessment

The preparation of the buffers, starch gel, PGI-2 detection, and phenotype assessment were performed according to Humphreys [31]. In brief, from each plant, about 0.2 g of young fresh leaves were crushed in a cooled mortar with 0.1 M TRIS, pH 7.2 extraction buffer. The filter paper wicks were soaked in the extract, the samples were placed on the starch gel (Sigma) for electrophoresis, and the gels were treated with the staining solution for PGI-2 detection and analysis.

### 2.3. Chromosome Preparation

The tillers collected from the plants were shortened, old roots were removed and set up in tap water in glass tubes for about 5–7 days, then fresh roots were collected and placed in ice-cold water for 24 h, followed by fixation in 1:3 acetic acid–ethanol. The roots were softened in a mixture of 0.1% pectolyase Y-23 and 0.1% cellulase R-10 and squashed in 45% acetic acid. Mitotic chromosome spreads were fixed on glass slides by freezing at −80 °C, coverslips removed and used fresh or stored at −20 °C. For the chromosome counts, ≥3 metaphases with a complete chromosome spread were analyzed in each individual. For GISH karyotyping, only the cells (3–5) with clear signals throughout the chromosome spreads were analyzed, and the most representative ones were chosen to be included in the Results and Supplementary data.

### 2.4. Fluorescent Probes

*L. multiflorum*, *F. pratensis* and *F. glaucescens* genomic DNA was sonicated for 5 min in an ultrasonic bath, checked on 1% agarose gel, and the DNA samples, sheared to 500–2500 bp fraction, were chosen for labelling. DNA was labelled with rhodamine-11-dUTP (Roche) or fluorescein-12-dUTP (Roche) by nick translation, using Nick Translation Mix kit (Roche). *L. multiflorum* rhodamine labelled probe was used specifically to highlight simultaneously both *L. multiflorum* (red) and *F. pratensis* (mauve), as described by Pašakinskienė et al. [30]. The pTA71 plasmid containing wheat 45S ribosomal DNA repeats was cleaved with the *EcoRI* restriction enzyme to release the insert and labelled with fluorescein-12-dUTP by nick translation.

### 2.5. In Situ Hybridization

The slides were soaked in 45% acetic acid for 5 min at room temperature, and at 48–50 °C for 3 min. Denaturation of genomic DNA was performed at 70 °C in 70% deionized formamide in 2xSSC for 2 min, followed by dehydration with cold ethanol series (70%, 90% and 100%), 2 min each, and air-drying. A 25 µL volume of hybridization mix made of about 100 ng of DNA probe, 60% formamide, 25% dextran sulfate, 10% 20xSSC, and 5% SDS was denatured at 70 °C for 10 min, then placed on slides and set up for incubation at 37 °C for 16 h in a moist chamber. After hybridization, the slides were washed in 20% formamide in 0.1xSSC twice at 42 °C for 5 min, and three times in 2xSSC at 42 °C for 3 min. The slides were mounted with Vectashield antifade and DAPI (4,6-diamidino-2-phenylindole) for DNA counterstaining. For reprobing with *F. glaucescens* genomic DNA and 45S rDNA probe, the *L. multiflorum* probe was washed off by soaking the slides in 4xSSC with Tween 20, the coverslips were removed, and the slides were washed three times in 2xSSC.

The slides with hybridized metaphase spreads were analyzed for hybridization signals under a Nikon Eclipse E800 fluorescence microscope. Three filter sets were used for detection: (i) DAPI (excitation 330–380 nm, beam 400 nm, barrier 420 nm); (ii) rhodamine (excitation 510–560 nm, beam 575 nm, barrier 590 nm); iii) fluorescein (excitation 450–490 nm, beam 505 nm; barrier 520 nm). Photographs were taken with a Pixera Penguin digital 600CL camera. Image Pro-Discovery 4.5 and Adobe Photoshop Elements were used for image processing.

## 3. Results

### 3.1. Somatic Tiller Chimerism in F2 3-18 Allohexaploid and Re-Establishment of Diploid Clonal Plants

**Figure 2:**
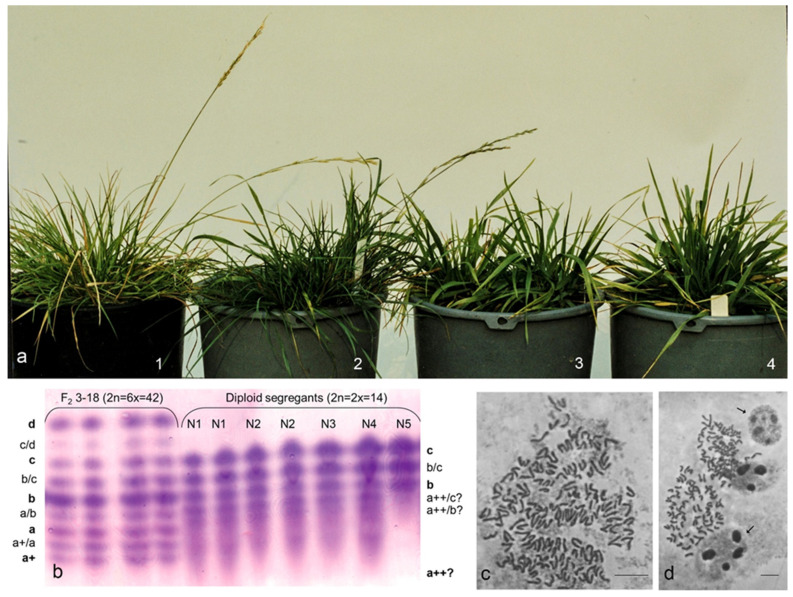
Phenotype view of F2 3-18 *L. multiflorum* × *F. arundinacea* donor and its split tiller decadents, PGI-2 isozyme pattern, and split anaphases in hyperploid cells. (**a**) 1—F2 3-18 donor plant (2n = 6x = 42); 2—identical clone (2n = 6x = 42), 3&4—diploid segregants (2n = 2x = 14). (**b**) PGI-2 isozyme separation, lanes 1–2 for the F2 3-18 donor plant, lanes 3–4 for the clones identical to donor, lanes 5–11 for diploid clonal plants, N1–5 labeled; F2 3-18 donor plant and its identical clones show a complex *a+*, *a*, *b*, *c*, and *d* allelic profile (true isoform bands for the PGI-2 alleles are labeled in bold, hybrid bands in regular font), N1–4 diploids show *a++bc*, N5—*bc* allele set. (**c**) Anaphases in hyperploid cell show several split division plates. (**d**) Metaphases with more than 42 chromosomes and nuclei of different sizes (arrowed) with variable nucleoli numbers and their sizes; chromosome counts are ca 163 in (**c**) and 68 in (**d**) (lower left). Scale bars = 10 and 20 µm in (**c**) and (**d**), respectively.

A peculiar “super-recombinant” genotype F2 3-18 was detected among 682 individuals from the cross *L. multiflorum* × *F. arundinacea* in F2C1 plant group screened morphologically. This genotype was multiplied clonally and showed a phenomenal effect of chimerism and emerging variable tiller phenotype (growth habit, leaf shape) different from that of the donor (Figure 1 and Figure 2a). Plants N1, N2, N4, and N5 had a typical inflorescence (panicle) characteristic of *F. pratensis*, while plant N3 never produced inflorescences. This particular instance of visually observed, distinctly different clonal segregants (Figure 2a) is the first case described in the *Festuca* × *Lolium* hybrid literature.

The somatic chromosome number of the F2 3-18 donor was determined as hexaploid 2n = 6x = 42 in the root meristematic cells cytologically examined. All five unusual plants, N1–N5, phenotypically contrasting with the donor plant, were determined as diploids (2n = 2x = 14), whereas the rest of the 22 clonal plants, phenotypically identical to the donor, remained hexaploid (2n = 6x = 42). Hyperploid nuclei, the split spindles’ anaphases and cells of different sizes were observed in the F2 3-18 donor (Figure 2c,d), which could presumably lead to heterogeneous cell populations in meristematic tissues.

Considering that the PGI-2 isozyme isoform profile is a unique and constant feature for a given plant, the F2 3-18 hybrid and clonal plants were analyzed for the PGI-2 phenotype. The PGI-2 profile in the F2 3-18 hybrid was found to be very complex, including all possible allelic isoforms, a^+^, a, b, c and d, whereas only b and c isoforms were clearly detected in the diploid segregants (Figure 2b). This pattern in the diploid segregants confirmed them as descendants of F2 3-18 and highlighted a tendency for a regular genome split. The distribution of the a^+^ isozyme fraction was not retained in the clonal diploids as it was in the original donor plant. Presumably, a^++^ instead of a^+^ was present in diploids, which explains the bands below b as the hybrid bands a^++^/c and a^++^/b. More importantly, the complexity of the pattern in four (N1–N4) out of five diploid segregants clearly shows them not being “plain” diploids since a maximum of two PGI-2 alleles are possible for diploids, e.g., *b* and *c* in a heterozygote. In other words, it is not possible to have three alleles in a diploid unless there is a third PGI-2 locus involved from the multiple gene set present in the donor hybrid. Taking into account the PGI-2 allele specificity throughout the parental species [32,42], these results point out that two components representing *F. glaucescens* had a different fate in the processes of rediploidization, namely *a^+^* appeared by its modified *a^++^* version, whereas the genome fraction carrying allele *d* was the one that was always left out.

Meiosis in the F3 3-18 plant was extremely irregular, with the multivalents of variable sizes and shapes prevalent in MI (Supplement Figure S1). Micronuclei, cytomixis and chromatin “bridges” were common in tetrads (Supplement Figure S1). Nevertheless, even though the meiosis view was not directly related to the discovery of somatic diploid descendants, it added data to nuclear content disruptions characteristic of the F2 3-18 donor plant.

### 3.2. GISH/FISH of Diploid Clonal Plants

**Figure 3:**
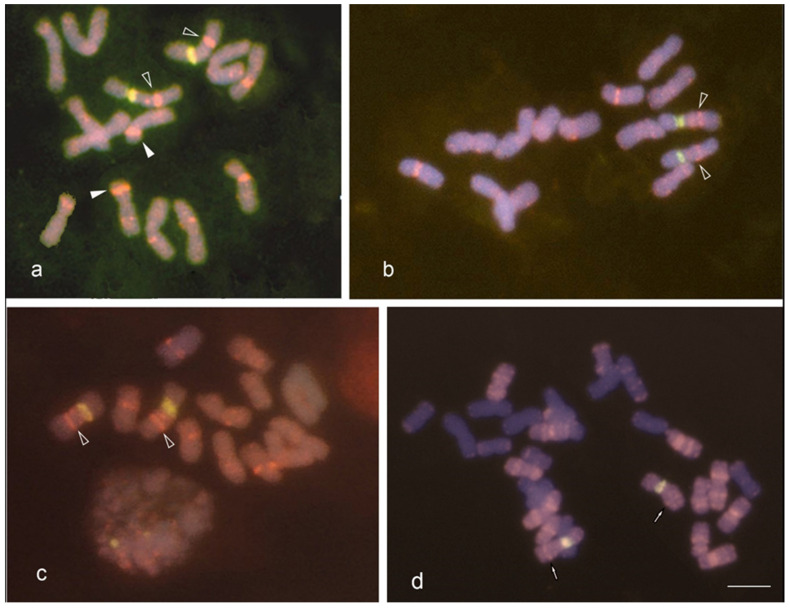
GISH/FISH in the metaphase plates of diploid (2n = 2x = 14) somatic segregants split out from the hexaploid F2 3-18 *L. multiflorum* × *F. arundinacea* plant (2n = 6x = 42). (**a**–**c**) Chromosome sets of diploids N1, N2 and N3 hybridized with *L. multiflorum* genomic DNA (red) and 45S rDNA probe (green) all show *F. pratensis*-like chromosomes (mauve) with some interstitial segments of *L. multiflorum* (red) on rDNA-bearing chromosomes (unfilled arrowheads), and on some other chromosomes (filled arrowheads), two 45S rDNA sites positioned near the centromere are visible in each chromosome set. (**d**) *F. pratensis* (mauve) subgenome chromosome set representation in *F. arundinacea* chromosome plate (incomplete), interstitial segments of *L. multiflorum* (red) on rDNA-bearing chromosomes marked by arrows; *F. glaucescens* subgenome chromosomes blue (DAPI). Scale bar = 10 µm.

*F. arundinacea* is an allohexaploid comprising two *Festuca* subgenomes, diploid *F. pratensis* and tetraploid *F. glaucescens*, defined as FpFpFgFgFgFg and having *L. multiflorum* introgressions within *F. pratensis* chromosomes [29,30]. GISH images of initial F1 and colchicine doubled hybrids are shown in the pedigree scheme (Figure 1). GISH analysis using an *L. multiflorum* genomic probe that simultaneously highlights *F. pratensis* and *L. multiflorum* chromosomes and chromosomal segments has shown diploid clonal plants to be new genomic variants. The karyotype of these newly split diploids was preferentially based on the *F. pratensis* subgenome, with *L. multiflorum* found in small variable-sized blocks (Figure 3a–c, Table 1). *F. glaucescens* segments were found as rare tiny insertions (image data not included). All of these *F. pratensis*-like diploids had two 45S rDNA sites at the interstitial position near the centromere on two chromosomes (Figure 3a–c, Table 1), which is a distinct feature of *F. pratensis* subgenome in *F. arundinacea* (Figure 3d).

Thus, GISH revealed the genome composition of the diploids and presented them as *F. pratensis*-like subgenome segregants established throughout somatic tiller chimerism. From these results, we can conclude that on the basis of genome irregularities that occurred in the particular somatically unstable allohexaploid *L. multiflorum* × *F. arundinacea* (2n = 6x = 42) F2 3-18 plant (one out of 682 in the F2 plant group), the diploid *F. pratensis* (2n = 2x = 14) subgenome of *F. arundinacea* was able to recompose and stabilize itself with minor footprints of other genomic partners, *L. multiflorum* and *F. glaucescens*.

### 3.3. GISH/FISH of F2 3-18 Super-Recombinant Donor

**Figure 4:**
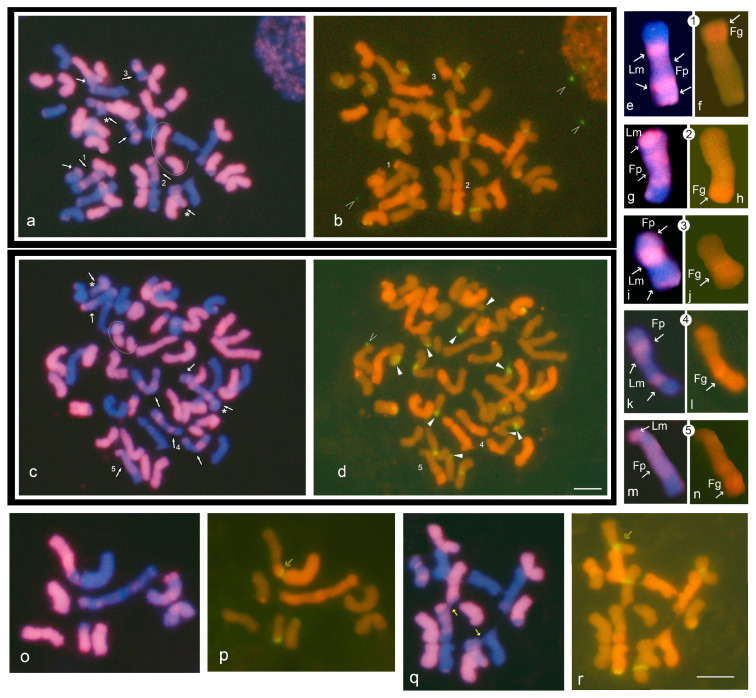
GISH/FISH in metaphase plates of *L. multiflorum* × *F. arundinacea* F2 3-18 donor (2n = 6x = 42) plant. Images of two representative mitotic metaphase chromosome spreads, A1 (**a**,**b**) and A2 (**c**,**d**), probed in two ways: (**a**,**c**) *L. multiflorum* genomic DNA probe detects *L. multiflorum* chromosomes and their segments in red and highlights *F. pratensis* in mauve, *F. glaucescens* is blue (DAPI); (**b**,**d**) metaphase spreads reprobed with genomic *F. glaucescens* DNA probe (orange) and 45S rDNA cluster sites hybridized by pTa71 probe appear in green; images are merged for red (rhodamine) and green (fluorescein). (**a**,**c**) *F. pratensis* chromosomal components (mauve) are the most involved in recombinant chromosomes; two-species recombinant chromosomes marked by bold arrows, three-species recombinant—by pointed arrows, pure *F. pratensis* chromosomes marked by an asterisk and arrowed; broken *L. multiflorum* chromosomes at the site of *F. pratensis* interstation are marked by circles. (**d**) In A2 metaphase spread, abundant broken sites appear at 45S rDNA positions (filled arrows); detached “floating” 45S rDNA pieces are visible ((**b**,**d**) open arrows). (**e**–**n**) Representation of several three-species, *F. pratensis*, *L. multiflorum*, and *F. glaucescens*, recombinant chromosomes; chromosome numbers correspond to those in (**a**–**d**). (**o**,**p**) Two irregular-shaped enlarged recombinant chromosomes depicted, 45S rDNA cluster at the junction point arrowed. (**q**,**r**) Sticking chromosome associations specified, protruding *F. pratensis* chromatin and rDNA-enriched site arrowed. Scale bar = 10 µm.

GISH/FISH was carried out to reveal genomic features of the donor *L. multiflorum* × *F. arundinacea* hybrid F2 3-18. The results are shown in Figure 4 for two representative mitotic chromosome spreads probed in two ways: (i) the total genomic DNA of *L. multiflorum*, which highlights both *L. multiflorum* (red) and *F. pratensis* (mauve) simultaneously (Figure 4a,c); (ii) the combined probe of total *F. glaucescens* DNA and pTa71 probe for 45S rDNA cluster (Figure 4b,d). In the F2 3-18 karyotype, *L. multiflorum* chromosomes and chromosomal parts were found to be the most prevalent (Figure 4a,c), followed by *F. glaucescens* (Figure 4b,d) and *F. pratensis* (Figure 4a,c), summarized in Table 2. About ¼ of the chromosomes were species-recombinant.

The types of recombinant chromosomes included all possible exchanges: doubles Lm/Fp, Fp/Fg, Lm/Fg, and triples Lm/Fp/Fg (Figure 4a–n, Table 2). GISH revealed a specific representation of *F. pratensis* in this hybrid karyotype, which appears clearly different from the representations of the other two species, *L. multiflorum* and *F. glaucescens*. Namely, *F. pratensis* chromosomal components were the least represented, but the most involved in chromosomal rearrangements (Figure 4a–d, Table 3).

The 45S rDNA clusters were abundant in the karyotype of the F2 3-18 plant. There were two different patterns of metaphases observed in the donor plant: (i) in general, chromosomes are intact, and there are few or no breakages (Figure 4a,b and Figure S2); (ii) chromosome breakages are abundant, predominantly at 45S rDNA positions of *L. multiflorum* chromosomes, and detached pieces of 45s DNA are present in and along the metaphase plate (Figure 4c,d, Figures S2 and S3). In contrast, 45S rDNA sites at the terminal position of two *F. glaucescens* chromosomes appeared to be stable (Figure 4b,d). Thus, a specific pronounced fragility at 45S rDNA positions in the F2 3-18 donor plant was observed with numerous freely floating detached 45S rDNA pieces in some metaphase plates (Figure 4b,d, Figures S2 and S3). This was a distinctive feature found in F23–18 metaphases, whereas no such patterns of scattered 45S rDNA components were observed in diploid metaphases (Figure 2a–c). When irregular-shaped enlarged recombinant chromosomes and sticking chromosome structures were analyzed (Figure 4o–r), it was found that some of them had a 45S rDNA block positioned at the junction point (Figure 4p). In addition, rDNA-enriched sites were seen to be involved in some sticking branched chromosome structures (Figure 4r).

Regarding visible *F. pratensis* chromatin disruptions, several *F. pratensis* chromatin protrusions at the sites of fusing chromosomal configurations (Figure 4q, Figure S2) and specific breakages at the position of *F. pratensis* insertion within *L. multiflorum* chromosomes were detected (Figure 4a,c).

The results of FISH/GISH analysis in this study show that a particularly irregular *L. multiflorum × F. arundinacea* F2 3-18 genotype is prone to complex chromosomal aberrations in the somatic tissues: abundance of recombinant chromosomes, chromosome fragility and stickiness, 45S rDNAs containing detached structures, and their involvement in chromosome fusing configurations (Figure 4, Figures S2 and S3). This suggests that recombinant chromosomes, with *F. pratensis* predominately involved, could be the source of vulnerable sites and could lead to extensive irregularities within the chromosome set of proliferating tissues in the plant body, which can finally turn out in split clonal plants of different ploidy, namely diploids (Figure 3).

**Figure 5:**
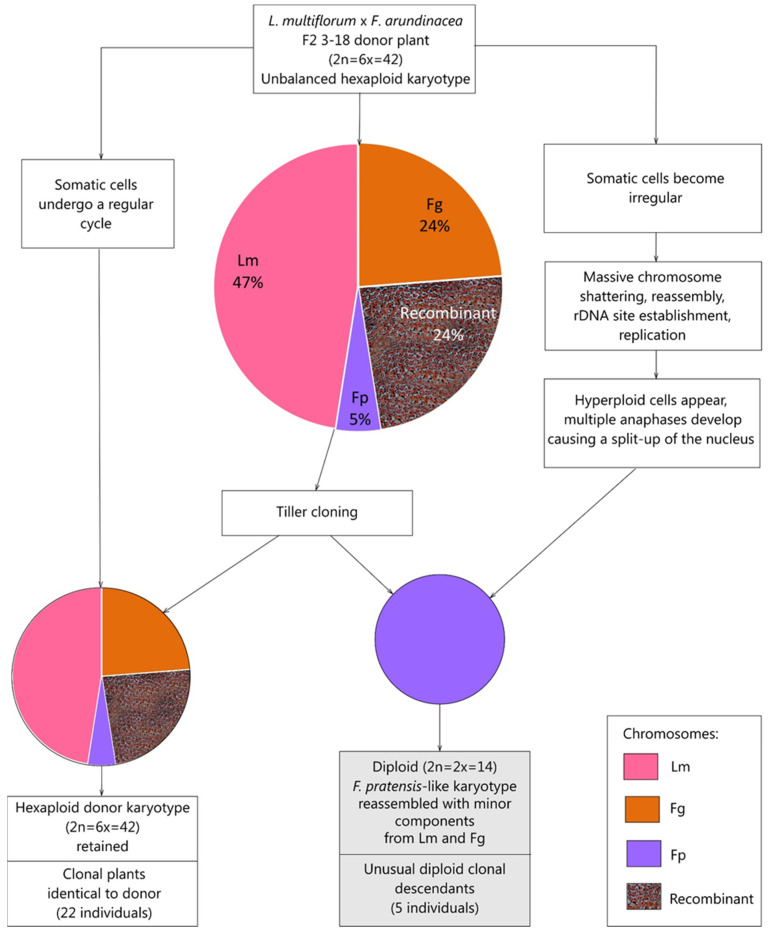
A proposed model of a diploid *F. pratensis*-like subgenome reassembly through a rare chromoanagenesis event in somatic tissues of *L. multiflorum* × *F. arundinacea* hybrid. Abbreviations: Lm—*L. multiflorum*, Fp*—F. pratensis*, Fg*—F. glaucescens* (Fp and Fg are subgenomes comprising *F. arundinacea* parent).

The reference data on the pronounced capability of *F. pratensis* for chromosome restructuring observed in *Festulolium* hybrids are summarized in Table 4. A hypothetical model of a diploid *F. pratensis*-like subgenome reassembly de novo is presented in Figure 5.

## 4. Discussion

Structural genomic changes in allopolyploids depending on the progenitor genomes were shown across different plant taxa by using GISH/FISH cytology and by sequence data assessment [3,11,16,46]. The breakage-fusion-bridge cycles may recompose the genome content in the somatic cells of the hybrids and account for the establishment of novel tiller phenotypes [47,48].

In the plethora of experimental plant hybrids, *Lolium* and *Festuca* species have been a very useful source to demonstrate how parental genomes adjust and rearrange themselves in intergeneric hybrids. In nearly three decades of FISH cytology studies, a wide spectrum of intergenomic adjustments has been determined in *Festulolium* hybrids. *Lolium* chromosomes dominate over *Festuca*; a high portion of chromosomes are species-recombinant; recombination events are significantly higher for *F. pratensis*-like chromosomes than for those of *Lolium*; 45S rRNA sites show high fragility; atypical interstitial positions of telomeric repeats appear in the hybrids [13,17,25,26,27,43,45,49]. In addition, a rare and more unusual case of instant rediploidization of the *F. pratensis* subgenome was described in F1C0 of the *L. multiflorum* × *F. arundinacea* (2n = 8x = 56) octoploid plant group [36].

Here, we follow up on the story of somatic chimerism and present a particularly complex case of genomic turbulence in the hexaploid genome of F2C1 *L. multiflorum × F. arundinacea* hybrid F2 3-18 (2n = 6x = 42), comprising *L. multiflorum* genome and two subgenomes of *F. arundinacea*, *F. pratensis* and *F. glaucescens*. In the first instance, this plant attracted attention for the distinct chimerism of its clonal parts, differing from the donor by their tiller phenotype (growth habit, leaf shape), and, cytologically, diploids. Furthermore, the screen for of PGI-2 isozyme phenotype clearly confirmed diploids to be descendants of the donor plant, with a regular tendency of a certain allele set retained in these somatic segregants. Cytological GISH/FISH analysis of the F2 3-18 donor genome presented us with a broad colorful canvas of a “chromosomal cocktail”. The recombinant chromosomes made up about 25% of the karyotype, and promiscuous exchange patterns between three genomes, *L. multiflorum*, *F. pratensis*, and *F. glaucescens*, were observed. Hence, it was rather unexpected to discover somatically segregating plants featuring a diploid (2n = 2x = 14) karyotype of a fairly defined genomic structure from the body of a donor hexaploid (2n = 6x = 42) plant representing such a chaotic “chromosomal cocktail”. Our findings show that all of these diploid clonal descendants have a few things in common: (i) the basic genome comes from *F. pratensis* progenitor subgenome with minor *L. multiflorum*, and, to a lesser extent, *F. glaucescens* components; (ii) they have two 45S rDNA sites positioned interstitially near the centromere, which corresponds to the variant of *F. pratensis* subgenome in *F. arundinacea*.

Admittedly, precise ways of the formation of *F. pratensis*-like diploids cannot be followed in time and space, and GISH/FISH analysis results are rather controversial. The split diploids are based on an *F. pratensis*-like genome, a minor component of the hybrid, whereas *L. multiflorum* and *F. glaucescens* chromosomes make up most of the donor plant genome. Therefore, some intriguing questions arise following a broader context of the genome conflict and adjustments in *Festulolium* hybrids. Why is it specifically the *F. pratensis* genome that splits out, “escapes” and re-establishes as diploid throughout vegetative tiller chimerism? An important point may be the involvement of 45S rDNA arrays and rDNA-bearing chromosomes [44,45]. Interestingly, the earlier studies by Sokolov et al. [50] on *Crepis capillaris* (Asteraceae) experimentally demonstrated that the presence of at least one NOR (45S rDNA) site-bearing chromosome in the karyotype is sufficient for the cell to be functional for replication. It is likely that due to genomic disturbance in the highly irregular karyotype of the *L. multiflorum* × *F. arundinacea* hybrid, the re-establishment and stabilization of the *F. pratensis* subgenome carrying two 45S rDNA sites [30] seems an easier task to be achieved than a *Lolium*-like variant with the abundant, 5–7, site number [51,52]. Likewise, it is quite possible that *Lolium* spp. chromosomes have an advantage and become dominant in *Lolium* x *Festuca* hybrids [25,26,27,43] exactly because of the abundance of their 45S rDNA clusters.

The results of this study together with observations of other research groups on *Festulolium* support the involvement of 45S rDNA loci in the karyotype rearrangements [17,26,45,53], (Table 4). More supportive data come from studies on other plant species [54,55,56,57]. Notably, similarly to our findings showing the involvement of rDNA-enriched clusters in the formation of sticking chromosome structures, Gernand et al. [58] recorded patterns in end-to-end and branching chromosomal associations in *Allium fistulosum* (Allioideae, Amaryllidaceae) triggered by tissue culture. Furthermore, the occurrence of detached rDNAs similar to ours was detected in *Tragopogon* species and their intergenic hybrids, suggesting that rDNAs display an active role in genome restructuring [59]. Whereas specifically, the intergenic spacer (IGS) within the 45S rDNA arrays was FISH labelled and traced as dynamic “carrier” of genomic blocks throughout the evolutionary changes of the *Senna tora* (Fabaceae) karyotype [60]. On the other hand, from the massive sequencing data, Long et al. [61] concluded that the 45S rDNA sequence amount is greatly variable, and in some *Arabidopsis thaliana* lines (out of 180 tested), this has resulted in a substantial increase in genome size. Thus, plant cytogenetics and DNA data studies show that rDNA arrays appear as nomadic genomic blocks capable of proliferation and may form some kind of intermediate self-organized structural elements in the process of genome rearrangements.

Even though GISH provides us with a highly informative picture of *L. multiflorum × F. arundinacea* donor plant genome composition and reveals the complexity of the chromosomal structures, the enigma of *F. pratensis*-like subgenome “escaping” from this “chromosomal cocktail” and rebuilding itself remains mysterious. Theoretically, there must have been short-lived moments of massive chromosomal breakages and reassembly that have little chance of being detected visually in the cells of a growing plant.

Recently, in the story of the plant genome restructuring through gross rearrangements of its structural building blocks, a new and striking phenomenon of chromosomal shattering and reassembly, for a long time only known in human cancer genetics [37,62,63], comes into light. In plants, the first cases have come from observations in woody plants where the major chromosome disruption and reassembly occurred spontaneously in clonal descendants or as a result of mutagenesis.

In Tempranillo Tinto grapevine, Carbonell-Bejerano et al. [40] applied the whole genome scale approach and discovered catastrophically unbalanced genome rearrangements caused by chromosomal shattering in a spontaneously arisen somatic clone showing the loss of berry color, named Tempranillo Blanco. This random reassembly involving the structural reshuffling of three chromosomes was defined as a case of chromoanagenesis (chromosome rebirth). Further on, short-read sequencing in *Populus* after gamma radiation mutagenesis demonstrated an instance of shattered chromosomes in two out of 592 individuals [41]. From these *Populus* lines, exhibiting extreme chromosomal restructuring, stable clones were produced and maintained vegetatively for several years. This shows that massive chromosome shattering and reassembly events can be tolerated with no severe effect on life. It is likely that such changes, although rare, may occur in the somatic tissues of long-lived plants, trees or perennial grasses, and may result in novel phenotypes bringing some advantages with the new set of traits.

Linking together the scientific outcomes of grapevine and *Populus* [40,41] and the patterns of genome restructuring in *Lolium* × *Festuca* hybrids [13,17,26,27,44,45,49], conclusions can be drawn about the evident differences between the species and a particular genotype in their ability for abrupt or/and massive chromosomal rearrangements. Meanwhile, it seems that *F. pratensis* chromosomes have a particular drive for chromosome restructuring [26,36,44,45]. There is something fundamental about the *F. pratensis* chromosome structure that prompts the disassembly/assembly process. Theoretically, besides rDNA arrays, the telomeric/centromeric repeats, and transposons involved, there could be some 3D properties of the chromatin that are still to be discovered.

In this study, a peculiar instance of *F. pratensis* subgenome reassembly and establishment was recorded as an outcome of the somatic tiller chimerism of an *L. multiflorum* × *F. arundinacea* F2C1 hybrid bearing a highly irregular karyotype. Though this phenomenon seems very rare, a similarly striking effect was noticed by Liv Østrem (pers. comm.) in another hybrid combination where a complete diploid *F. pratensis* (2n = 2x = 14) surprisingly re-emerged in the cross *L. perenne* × (*L. perenne* × *Festulolium* [var. Prior]) (2n = 4x = 28). Remarkably, there was no full set of *F. pratensis* involved in the first place, as it is known to be represented by only 3.5 (out of 14) chromosomes in the plants of var. Prior [43]. This observation and our results showing *F. pratensis* rebuilding itself from the minor component of the hybrid suggest the involvement of structural resources from the bulk of two/three-species recombinant chromosomes. At the moment, it is difficult to imagine how exactly such a scenario could be governed and accomplished in the cell. Our results show that 45S rDNA-containing structures are likely to be dynamic intermediates involved in de novo *F. pratensis*-like chromosome rebuilding. Notably, rDNA resources were highly abundant both in the donor F2 3-18 karyotype and in the cross-combination *L. perenne* × (*L. perenne* × *Festulolium* [var. Prior]). Therefore, the abundance of rDNA seems to be a key condition for chromoanagenesis of *F. pratensis*-like karyotype from the complex “chromosomal cocktail” in the hybrid.

The extreme plasticity of the genomes, occurring through chromosome shattering and chromoanagenesis, and its significance in macroevolution has been well reviewed by Pellestor and co-authors [38,39], pointing towards the genome-centric (as opposed and complementary to gene-centric) approach in building up the full picture of speciation processes in eukaryotes. In plants, some ambiguous aberrant chromosomal structures detected by FISH in *A. fistulosum* tissue culture-derived plants [58] and extrachromosomal circular DNAs maintained in the herbicide-resistant weed *Amaranthus palmeri* (Amaranthaceae) [64] extend our knowledge of surprising ways of genome self-reorganizing responses and point to avenues of new discoveries. Very recently, Majka and co-authors [65] provided new insights into the mechanism of perpetual pushing around and competition within the *Lolium* × *Festuca* hybrid genome with an in-depth study of parental chromosome behavior at the meiosis level.

Since the work of Barbara McClintock [66], there are things still to be discovered about jumping genes and recombining genome components in a non-Mendelian way. This study demonstrates an instance of a mysterious reassembly (“rebirth”) of the *F. pratensis*-like genome, which adds to the growing data proving the existence of sudden genomic alterations beyond genes. This comes in line with the intriguing chromoanagenesis phenomena and highlights the role of gross abrupt genome changes in the evolutionary processes of plant taxa [67]. In practical terms, such spontaneous genome-scale somatic variability may provide a new and unexpected set of traits in herbaceous and woody plants.

## Figures and Tables

**Table 1:** Summarized data on the genome composition of the clonal diploid segregants (2n = 2x = 14) from the hexaploid F2 3-18 *L. multiflorum* × *F. arundinacea* (2n = 6x = 42) hybrid.

Clonal Diploid Plant	Parental Genome/Subgenome Proportion According to GISH *			45S rDNA Cluster Site Number	Presence of PGI-2 Allele a^++^ from Fg Subgenome **
	Major Chromosomal Component	Lm Insertions	Fg Insertions		
N1	Fp	++	−	2	Yes
N2	Fp	+	−	2	Yes
N3	Fp	+	+?	2	Yes
N4	Fp	++	+?	2	Yes
N5	Fp	+	−	2	No

* The degree of parental genome/subgenome presence in the clonal diploids is indicated from GISH results by increasing number +; the lack of hybridization is indicated by −. ** Allele a++ is defined as a modified version of a+ deriving from *F. glaucescens* subgenome of the donor plant F2 3-18.

**Table 2:** Genomic complexity of super-recombinant F2 3-18 *L. multiflorum* × *F. arundinacea* revealed by GISH for its true parental and recombinant chromosomal components (structural analysis from Figure 4).

Parental Chromosomes and a Category of Recombinant Chromosome Variant	Metaphase Spread	
	A1	A2
Lm chromosomes and chromosomal parts	20	24
Ratio of broken/total Lm	1/20	8/24
Lm + Fp tip	1	2
⅔Lm + ⅓Fp	2	2
⅔Lm + ⅓Fg	1	2
Fg chromosomes	10	10
Fg +Fp tip	2	2
⅔Fg + ⅓Fp	1	1
½Fg + ¼Lm + ¼Fp	2	2
Fp chromosomes	2	2
¾Fp + 2 Lm inserts + ¼Fg	1	1
½Fp + ¼Lm+ ¼Fg	1	1

**Table 3:** The proportion of parental species represented in the recombinant chromosomes of donor plant F2 3-18 *L. multiflorum* × *F. arundinacea*.

Parental Species Genome or Subgenome *	Chromosomes Having Full or Partial Species Components	Recombinant Chromosomes **	
		No.	%
*L. multiflorum*	119	35	29,4
*F. glaucescens*	68	28	41,2
*F. pratensis*	46	36	78,3

* *F. glaucescens* and *F. pratensis* are subgenomes comprising *F. arundinacea* parent. ** Data from chromosome analysis in metaphase spreads in Figure 4a–d, and in Supplement Figure S2.

**Table 4:** Summarized data on *F. pratensis* apparent feature for marked chromosomal structural changes in *Festulolium* hybrids.

Reference Source	*Festulolium* Hybrids	Description
Pašakinskienė et al., 1997 [36]	*L. multiflorum* × *F. arundinacea* (2n = 8x = 56) F1 plants	An unusual case of instant rediploidization of the *F. pratensis* subgenome was discovered; six “novel diploids” were found out of 167 in the F1C0 octoploid plant group.
Canter et al., 1999 [43]	*L. perenne* × *F. pratensis* var. Prior (2n = 4x = 28) plants in F8 generation	Recombination events per recombined chromosome were significantly higher for *F. pratensis* than for *L. perenne*-like chromosomes.
Zwierzykowsky et al., 2006 [26]	*F. pratensis* × *L. perenne* (2n = 4x = 28) plants in six successive generations F1–F6	The number of recombinant chromosomes and recombination break points were higher for *F. pratensis* than for *L. perenne*-like chromosomes.
Książczyk et al., 2015 [44]	*F. pratensis* × *L. perenne* plants (2n = 4x = 28) in F2–F4 generations	*F. pratensis*-like chromosomes are less structurally stable than *L. perenne*; *F. pratensis*-like chromosomes were more affected by rDNA loci changes.
Majka et al., 2018 [17]	*F. pratensis* × *L. perenne* plants (2n = 4x = 28) in F1–F9 generations	An unusual variant of the rDNA cluster site in a pair of *F. pratensis* chromosomes was detected, with two loci found on one chromosome and one on another.
Majka et al., 2019 [45]	*F. pratensis* × *L. perenne* plants (2n = 4x = 28) in F2–F3 generations	Cytogenetic and molecular genotyping (ISSR) revealed higher levels of recombination in chromosomes derived from *F. pratensis* than from *L. perenne*; structural changes were more frequent in *F. pratensis*-like chromosomes bearing 45S rDNA loci than in non-bearing ones.
Liv Østrem (personal communication), 2002	*L. perenne* × (*L. perenne* × *Festulolium* [var. Prior]) (2n = 4x = 28) backcross plants	A complete diploid *F. pratensis* genome (2n = 2x = 14) surprisingly re-emerged in the cross.
This study	A unique *L. multiflorum* × *F. arundinacea* (2n = 6x = 42) “super-recombinant” donor plant showing distinct clonal tiller variability found among 682 plants in F2	Reassembly of the *F. pratensis* subgenome was discovered from a “chromosomal cocktail” represented in a hexaploid donor plant; five “novel diploids” were found out of 27 clonal descendants.

## Data Availability

All of the data are included in the main text.

## Supplementary Materials

The following supporting information can be downloaded at: https://www.mdpi.com/article/10.3390/plants12050984/s1, Figure S1: Chromosome pairing structures in MI of F2 3-18 plant; Figure S2: Extra GISH/FISH metaphase plates of F2 3-18 donor plant. Figure S3: Extended GISH/FISH view of metaphase plate of F2 3-18 donor specifying the patterns of detached “floating” 45S rDNAs.

## References

[1] Whitney K.D., Ahern J.R., Campbell L.G., Albert L.P., King M.S. (2010). Patterns of hybridization in plants. Perspect. Plant Ecol. Evol. Syst..

[2] Barker M.S., Arrigo N., Baniaga A.E., Li Z., Levin D.A. (2016). On the relative abundance of autopolyploids and allopolyploids. New Phytol..

[3] Soltis D.E., Visger C.J., Marchant D.B., Soltis P.S. (2016). Polyploidy: Pitfalls and paths to a paradigm. Am. J. Bot..

[4] Renny-Byfield S., Wendel J.F. (2014). Doubling down on genomes: Polyploidy and crop plants. Am. J. Bot..

[5] Salse J. (2016). Ancestors of modern plant crops. Curr. Opin. Plant Biol..

[6] Schiessl S.-V., Katche E., Ihien E., Chawla H.S., Mason A.S. (2018). The role of genomic structural variation in the genetic improvement of polyploid crops. Crop J..

[7] Liu B., Wendel J.F. (2002). Non-mendelian phenomena in allopolyploid genome evolution. Curr. Genom..

[8] Leitch I.J., Bennett M.D. (2004). Genome downsizing in polyploid plants. Biol. J. Linn. Soc..

[9] Jones N., Pašakinskienė I. (2005). Genome conflict in Gramineae. New Phytol..

[10] Escudero M., Martín-Bravo S., Mayrose I., Fernández-Mazuecos M., Fiz-Palacios O., Hipp A.L., Pimentel M., Jiménez-Mejías P., Valcárcel V., Vargas P. (2014). Karyotypic changes through dysploidy persist longer over evolutionary time than polyploid changes. PLoS ONE.

[11] Edger P.P., Smith R., McKain M.R., Cooley A.M., Vallejo-Marin M., Yuan Y., Bewick A.J., Ji L., Platts A.E., Bowman M.J. (2017). Subgenome dominance in an interspecific hybrid, synthetic allopolyploid, and a 140-year-old naturally established neo-allopolyploid monkeyflower. Plant Cell.

[12] Kalinka A., Achrem M. (2018). Reorganization of wheat and rye genomes in octoploid triticale (× *Triticosecale*). Planta.

[13] Glombik M., Copetti D., Bartos J., Stoces S., Zwierzykowski Z., Ruttink T., Wendel J.F., Duchoslav M., Dolezel J., Studer B. (2021). Reciprocal allopolyploid grasses (*Festuca* × *Lolium*) display stable patterns of genome dominance. Plant J..

[14] Bird K.A., Van Buren R., Puzey J.R., Edger P.P. (2018). The causes and consequences of subgenome dominance in hybrids and recent polyploids. New Phytol..

[15] Alger E.I., Edger P.P. (2020). One subgenome to rule them all: Underlying mechanisms of subgenome dominance. Curr. Opin. Plant Biol..

[16] Murat F., Xu J.H., Tannier E., Abrouk M., Guilhot N., Pont C., Messing J., Salse J. (2010). Ancestral grass karyotype reconstruction unravels new mechanisms of genome shuffling as a source of plant evolution. Genome Res..

[17] Majka J., Zwierzykowski Z., Majka M., Kosmala A. (2018). Karyotype reshufflings of *Festuca pratensis* × *Lolium perenne* hybrids. Protoplasma.

[18] Wu Y., Lin F., Zhou Y., Wang J., Sun S., Wang B., Zhang Z., Li G., Lin X., Wang X. (2021). Genomic mosaicism due to homoeologous exchange generates extensive phenotypic diversity in nascent allopolyploids. Natl. Sci. Rev..

[19] Bashir T., Mishra R.C., Hasan M.M., Mohanta T.K., Bae H. (2018). Effect of hybridization on somatic mutations and genomic rearrangements in plants. Int. J. Mol. Sci..

[20] Schoen D.J., Schultz S.T. (2019). Somatic mutation and evolution in plants. Annu. Rev. Ecol. Evol. Syst..

[21] Piperidis G., Piperidis N., D’Hont A. (2010). Molecular cytogenetic investigation of chromosome composition and transmission in sugarcane. Mol. Genet. Genom..

[22] Winterfeld G., Becher H., Voshell S., Hilu K., Röser M. (2018). Karyotype evolution in *Phalaris* (Poaceae): The role of reductional dysploidy, polyploidy and chromosome alteration in a wide-spread and diverse genus. PLoS ONE.

[23] Malik C.P., Thomas P.T. (1966). Karyotypic studies in some *Lolium* and *Festuca* species. Caryologia.

[24] Catalan P., Torrecilla P., Rodriguez J.A.L., Olmstead R.G. (2004). Phylogeny of the festucoid grasses of subtribe Loliinae and allies (Poeae, Pooideae) inferred from ITS and trnL-F sequences. Mol. Phylogenet. Evol..

[25] Kopecký D., Loureiro J., Zwierzykowski Z., Ghesquiere M., Dolezel J. (2006). Genome constitution and evolution in *Lolium* × *Festuca* hybrid cultivars (*Festulolium*). Theor. Appl. Genet..

[26] Zwierzykowski Z., Kosmala A., Zwierzykowska E., Jones N., Joks W., Bocianowski J. (2006). Genome balance in six successive generations of the allotetraploid *Festuca pratensis* × *Lolium perenne*. Theor. Appl. Genet..

[27] Lideikytė L., Pašakinskienė I. (2007). Genomic composition of amphiploid × *Festulolium braunii* cultivars ‘Punia’ and ‘Rakopan’. Zemdirbyste-Agriculture.

[28] Humphreys M.W., Zwierzykowski Z. (2020). *Festulolium*, a century of research and breeding and its increased relevance in meeting the requirements for multifunctional grassland agriculture. Biol. Plant..

[29] Humphreys M.W., Thomas H.M., Morgan W.G., Meredith M.R., Harper. J.A., Thomas H., Zwierzykowski Z., Ghesquiére M. (1994). Discriminating the ancestral progenitors of hexaploid *Festuca arundinacea* using genomic *in situ* hybridization. Heredity.

[30] Pašakinskienė I., Anamthawat-Jónsson K., Humphreys M.W., Paplauskienė V., Jones R.N. (1998). New molecular evidence on genome relationships and chromosome identification in *Festuca* and *Lolium*. Heredity.

[31] Humphreys M.W. (1989). The controlled introgression of *Festuca arundinacea* genes into *Lolium multiflorum*. Euphytica.

[32] Humphreys M.W., Ghesquiere M. (1994). Assessing success in gene transfer between *Lolium multiflorum* and *Festuca arundinacea*. Euphytica.

[33] Kleijer G. (1987). Cytogenetic studies of crosses between *Lolium multiflorum* Lam. and *Festuca arundinacea* Schreb. III. The generations C1, C2 and C3. Plant Breed..

[34] Humphreys M.W., Zare A.G., Pašakinskienė I., Thomas H., Rogers W.J., Collin H.A. (1998). Interspecific genomic rearrangements in androgenic plants derived from a *Lolium multiflorum* × *Festuca arundinacea* (2n = 5x = 35) hybrid. Heredity.

[35] Akiyama Y., Ueyama Y., Hamada S., Kubota A., Kato D., Yamada-Akiyama H., Takahara Y., Fujimori M. (2016). Utilization of flow cytometry for *Festulolium* breeding (*Lolium multiflorum* (2x) × *Festuca arundinacea* (6x)). Breed. Sci..

[36] Pašakinskienė I., Anamthawat-Jónsson K., Humphreys M.W., Jones R.N. (1997). Novel diploids following chromosome elimination and somatic recombination in *Lolium multiflorum* × *Festuca arundinacea* hybrids. Heredity.

[37] Holland A.J., Cleveland D.W. (2012). Chromoanagenesis and cancer: Mechanisms and consequences of localized, complex chromosomal rearrangements. Nat. Med..

[38] Pellestor F., Gatinois V. (2020). Chromoanagenesis: A piece of the macroevolution scenario. Mol. Cytogenet..

[39] Pellestor F., Gaillard J.B., Schneider A., Puechberty J., Gatinois V. (2022). Chromoanagenesis, the mechanisms of a genomic chaos. Semin. Cell Dev. Biol..

[40] Carbonell-Bejerano P., Royo C., Torres-Pérez R., Grimplet J., Fernandez L., Franco-Zorrilla Lijavetzky D., Baroja E., Martínez J., García-Escudero E., Ibáñez J. (2017). Catastrophic unbalanced genome rearrangements cause somatic loss of berry color in grapevine. Plant Physiol..

[41] Guo W., Comai L., Henry I.M. (2021). Chromoanagenesis from radiation-induced genome damage in *Populus*. PLoS Genet..

[42] Humphreys M.W., Thomas H.M., Harper J., Morgan G., James A., Ghamari-Zare A., Thomas H. (1997). Dissecting drought-and cold-tolerance traits in the *Lolium*-*Festuca* complex by introgression mapping. New Phytol..

[43] Canter P.H., Pašakinskienė I., Jones R.N., Humphreys M.W. (1999). Chromosome substitutions and recombination in the amphiploid *Lolium perenne* × *Festuca pratensis* cv. Prior (2n = 4x = 28). Theor. Appl. Genet..

[44] Książczyk T., Zwierzykowska E., Molik K., Taciak M., Krajewski P., Zwierzykowski Z. (2015). Genome-dependent chromosome dynamics in three successive generations of the allotetraploid *Festuca pratensis* × *Lolium perenne* hybrid. Protoplasma.

[45] Majka J., Bzdęga K., Janiak A., Ćwiek-Kupczyńska H., Krajewski P., Książczyk T., Zwierzykowski Z. (2019). Cytogenetic and molecular genotyping in the allotetraploid *Festuca pratensis* × *Lolium perenne* hybrids. BMC Genom..

[46] Nieto-Feliner G., Casacuberta J., Wendel J.F. (2020). Genomics of evolutionary novelty in hybrids and polyploids. Front. Genet..

[47] Schubert I., Lysak M.A. (2011). Interpretation of karyotype evolution should consider chromosome structural constraints. Trends Genet..

[48] Danilova T.V., Akhunova A.R., Akhunov E.D., Friebe B., Gill B.S. (2017). Major structural genomic alterations can be associated with hybrid speciation in *Aegilops markgrafii* (Triticeae). Plant J..

[49] Kopecký D., Horáková L., Duchoslav M., Doležel J. (2019). Selective elimination of parental chromatin from introgression cultivars of x*Festulolium* (*Festuca* × *Lolium*). Sustainability.

[50] Sokolov N.N., Sidorov B.N., Durimanova S.A. (1974). Genetic control of DNA replication in chromosomes of eukaryotes. Theor. Appl. Genet..

[51] Thomas H.M., Harper J.A., Meredith M.R., Morgan W.G., Thomas I.D., Timms E., King I.P. (1996). Comparison of ribosomal DNA sites in *Lolium* species by fluorescence in situ hybridization. Chromosome Res..

[52] Lideikytė L., Pašakinskienė I., Lemežienė N., Nekrošas S., Kanapeckas J. (2008). FISH assessment of ribosomal DNA sites in the chromosome sets of *Lolium*, *Festuca* and *Festulolium*. Zemdirbyste-Agriculture.

[53] Thomas H.M., Harper J.A., Morgan W.G. (2001). Gross chromosome rearrangements are occurring in an accession of the grass *Lolium rigidum*. Chromosome Res..

[54] Raskina O., Barber J.C., Nevo E., Belyayev A. (2008). Repetitive DNA and chromosomal rearrangements: Speciation-related events in plant genomes. Cytogenet. Genome Res..

[55] Garcia S., Kovarik A. (2013). Dancing together and separate again: Gymnosperms exhibit frequent changes of fundamental 5S and 35S rRNA gene (rDNA) organisation. Heredity.

[56] Sousa A., Renner S.S. (2015). Interstitial telomere-like repeats in the monocot family Araceae. Bot. J. Linn. Soc..

[57] Havlová K., Dvořáčková M., Peiro R., Abia D., Mozgová I., Vansáčová L., Gutierrez C., Fajkus J. (2016). Variation of 45S rDNA intergenic spacers in *Arabidopsis thaliana*. Plant Mol. Biol..

[58] Gernand D., Golczyk H., Rutten T., Ilnicki T., Houben A., Joachimiak A.J. (2007). Tissue culture triggers chromosome alterations, amplification and transposition of repeat sequences in *Allium fistulosum*. Genome.

[59] Kovarik A., Pires J.C., Leitch A.R., Lim K.Y., Sherwood A.M., Matyasek R., Rocca J., Soltis D.E., Soltis P.S. (2005). Rapid concerted evolution of nuclear ribosomal DNA in two *Tragopogon* allopolyploids of recent and recurrent origin. Genetics.

[60] Waminal N.E., Pellerin R.J., Kang S.-H., Kim H.H. (2021). Chromosomal mapping of tandem repeats revealed massive chromosomal rearrangements and insights into *Senna tora* dysploidy. Front. Plant. Sci..

[61] Long Q., Rabanal F.A., Meng D., Huber C.D., Farlow A., Platzer A., Zhang Q., Vilhjálmsson B.J., Korte A., Nizhynska V. (2013). Massive genomic variation and strong selection in *Arabidopsis thaliana* lines from Sweden. Nat. Genet..

[62] Forment J.V., Kaidi A., Jackson S.P. (2012). Chromothripsis and cancer: Causes and consequences of chromosome shattering. Nat. Rev. Cancer.

[63] Shen M.M. (2013). Chromoplexy: A new category of complex rearrangements in the cancer genome. Cancer Cell.

[64] Koo D.-H., Molin W.T., Saski C.A., Jiang J., Putta K., Jugulam M., Friebe B., Gill B.S. (2018). Extrachromosomal circular DNA-based amplification and transmission of herbicide resistance in crop weed *Amaranthus palmeri*. Proc. Natl. Acad. Sci. USA.

[65] Majka J., Glombik M., Doležalová A., Kneřová J., Ferreira M.T.M., Zwierzykowski Z., Duchoslav M., Studer B., Doležel J., Bartoš J. (2023). Both male and female meiosis contribute to non-Mendelian inheritance of parental chromosomes in interspecific plant hybrids (*Lolium* × *Festuca*). New Phytol..

[66] McClintock B. (1984). The significance of responses of the genome to challenge. Science.

[67] Mandáková T., Pouch M., Brock J.R., Al-Shehbaz I.A., Lysak M.A. (2019). Origin and evolution of diploid and allopolyploid *Camelina* genomes were accompanied by chromosome shattering. Plant Cell.

