# Supplementary material for: Festuca pratensis-like Subgenome Reassembly from a “Chromosomal Cocktail” in the Intergeneric Festulolium (Poaceae) Hybrid: A Rare Chromoanagenesis Event in Grasses
Source: Plants (Basel). 2023 Feb 21;12(5):984. doi: 10.3390/plants12050984 (PMC10005718; doi:10.3390/plants12050984)

Figure S1. Meiosis patterns of F2 3-18 plant. (a,b) Chromosome pairing structures in MI with the multivalents of variable sizes and irregular shapes prevalent; (c,d) micronuclei, cytomixis, stickiness and chromatin bridges in tetrads. Scale bars 10 and 60 µm, in a and c respectively.

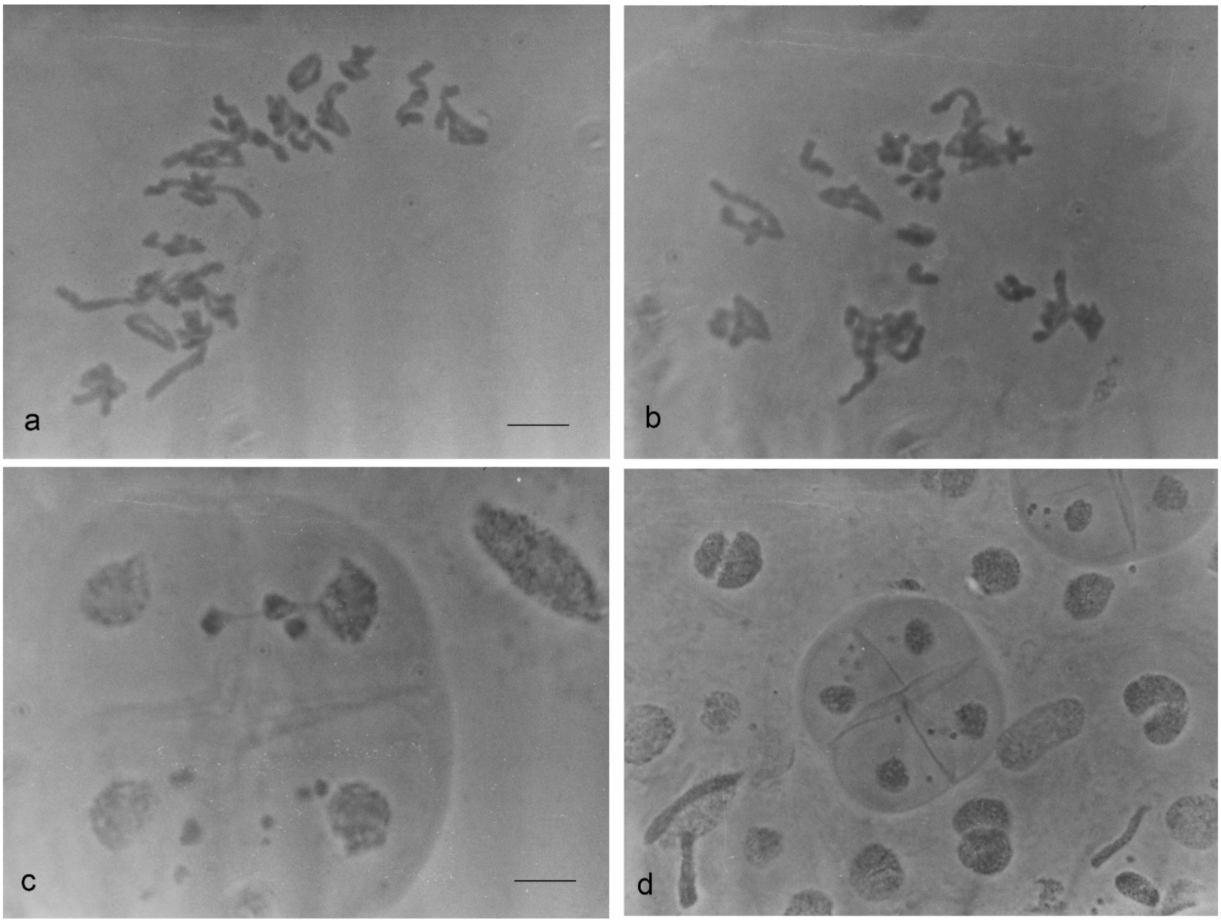

Supplement: Supplementary file 1 [file plants-12-00984-s001.zip › Supplement S1.pdf]
