# Supplementary material for: Festuca pratensis-like Subgenome Reassembly from a “Chromosomal Cocktail” in the Intergeneric Festulolium (Poaceae) Hybrid: A Rare Chromoanagenesis Event in Grasses
Source: Plants (Basel). 2023 Feb 21;12(5):984. doi: 10.3390/plants12050984 (PMC10005718; doi:10.3390/plants12050984)

Figure S2. Two extra GISH/FISH metaphase plates of *L. multiflorum* x *F. arundinacea* F2 3-18 donor (2n=6x=42) plant. Images of the chromosome metaphase spreads, probed in two ways: (**a,c**) *L. multiflorum* in red and *F. pratensis* in mauve, *F. glaucescens* is blue (DAPI); (**b,d**) metaphase spreads reprobed detecting *F. glaucescens* in orange, and 45S rDNA cluster sites in green; images are merged for red (rhodamine) and green (fluorescein). 45S rDNA signals are abundant in b,d; in b, several sites of 45S rDNA are broken, resulting in split chromosomes and a derivative microstructure containing 45S rDNA is indicated by an arrow; in d, 45S rDNA sites and chromosomes remain intact; in c sticking chromosomes involving *F. pratensis* chromatin marked by an arrow. Scale bar = 10 μm.

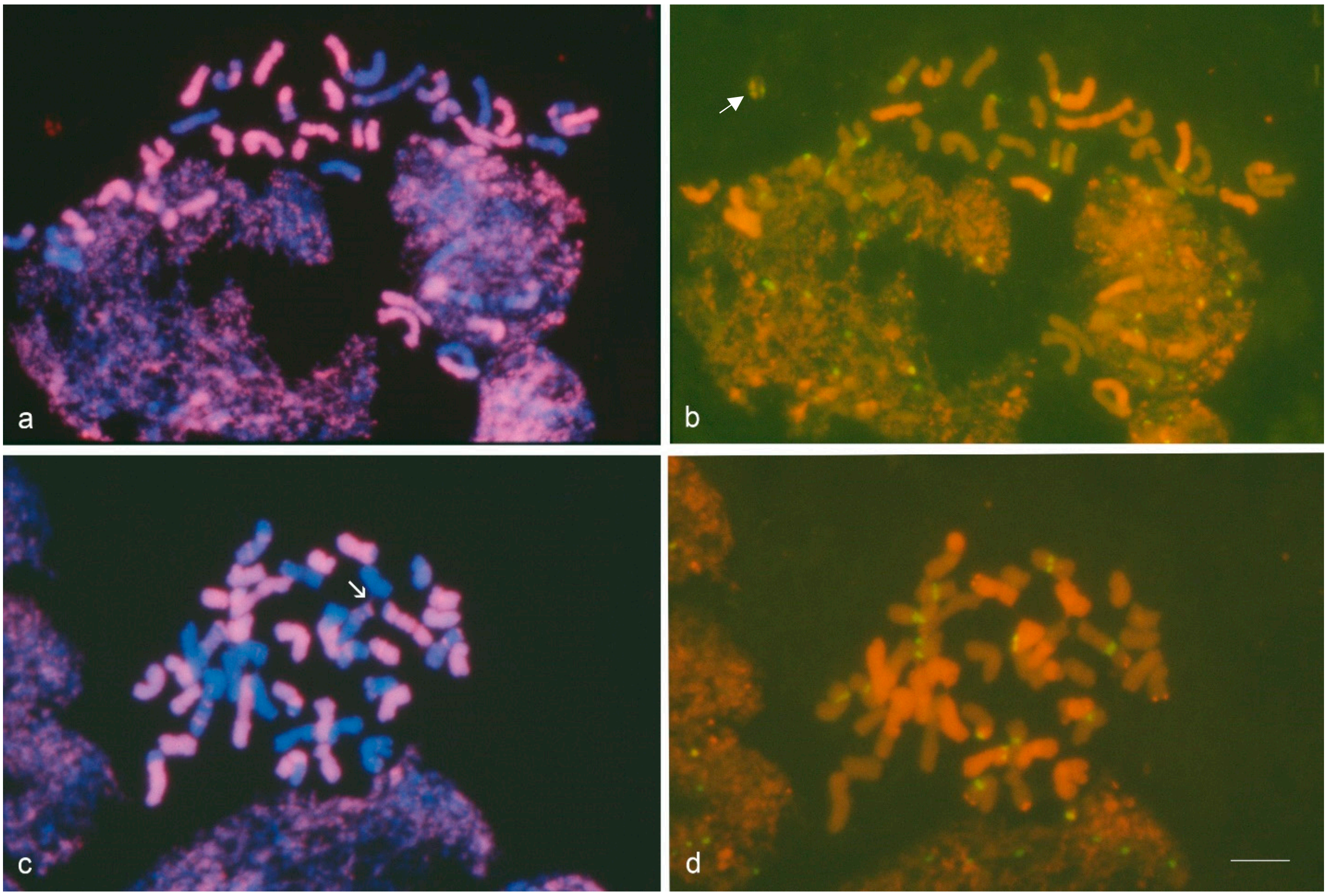

Supplement: Supplementary file 1 [file plants-12-00984-s001.zip › Supplement S2.pdf]
