# Supplementary material for: Festuca pratensis-like Subgenome Reassembly from a “Chromosomal Cocktail” in the Intergeneric Festulolium (Poaceae) Hybrid: A Rare Chromoanagenesis Event in Grasses
Source: Plants (Basel). 2023 Feb 21;12(5):984. doi: 10.3390/plants12050984 (PMC10005718; doi:10.3390/plants12050984)

Figure S3. Extended view of the GISH/FISH metaphase image (corresponds to Fig. 4 a,b) specifying the patterns of detached "floating" 45S rDNAs in and along metaphase plate of F2 3-18 donor plant; numerous detached 45S rDNAs are arrowed in b. Scale bar = 10 µm.

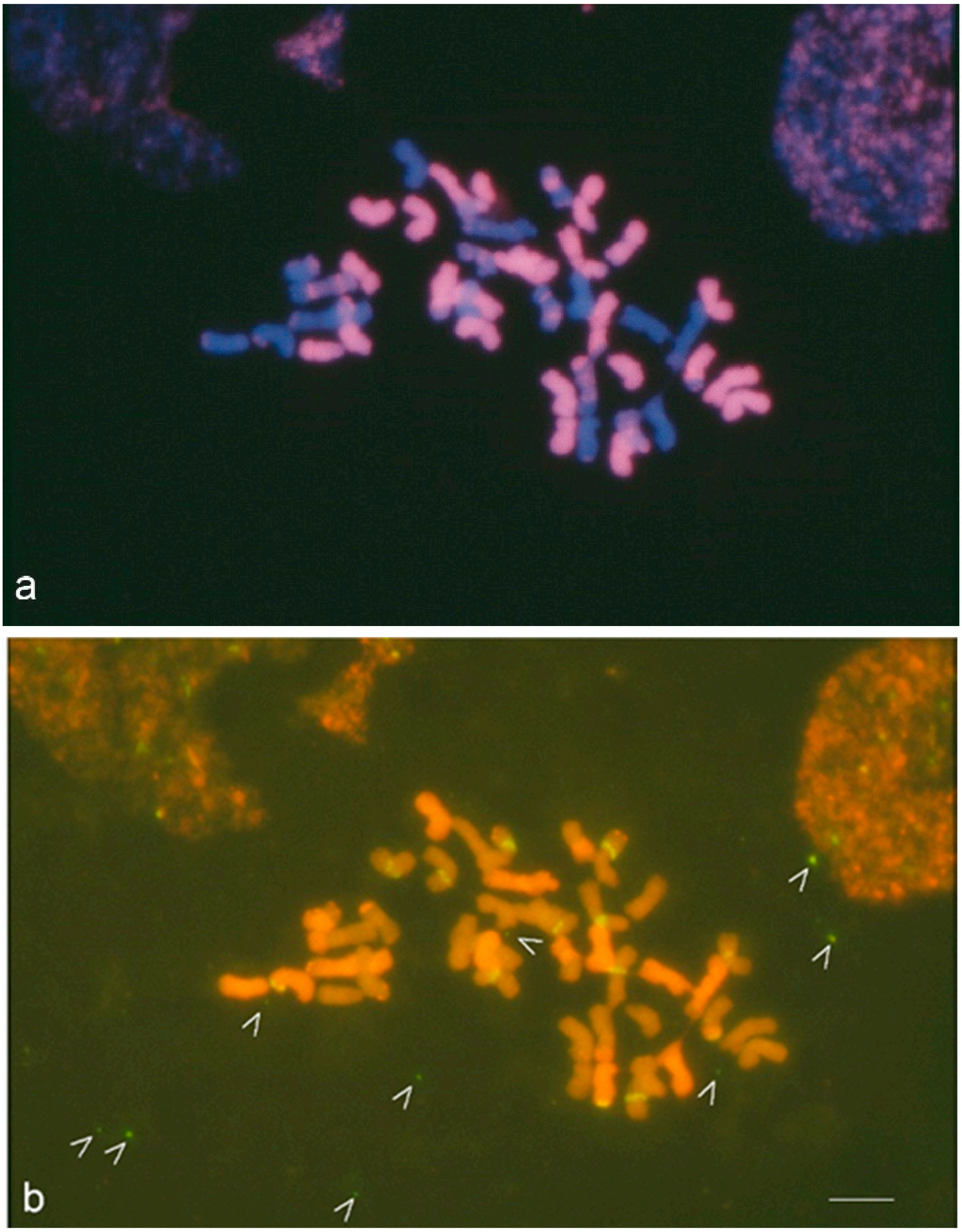

Supplement: Supplementary file 1 [file plants-12-00984-s001.zip › Supplement S3.pdf]
